# Natural immunity stimulation using ELICE16INDURES® plant conditioner in field culture of soybean

**DOI:** 10.1016/j.heliyon.2023.e12907

**Published:** 2023-01-10

**Authors:** Kincső Decsi, Barbara Kutasy, Géza Hegedűs, Zoltán Péter Alföldi, Nikoletta Kálmán, Ágnes Nagy, Eszter Virág

**Affiliations:** a Department of Plant Physiology and Plant Ecology, Campus Keszthely, Hungarian University of Agriculture and Life Sciences Georgikon, Keszthely, Hungary; b EduCoMat Ltd., Keszthely, Hungary; c Department of Information Technology and Its Applications, Faculty of Information Technology, University of Pannonia, Zalaegerszeg, Hungary; d Institute of Metagenomics, University of Debrecen, Debrecen, Hungary; e Department of Environmental Biology, Campus Keszthely, Hungarian University of Agriculture and Life Sciences Georgikon, Keszthely, Hungary; f Department of Biochemistry and Medical Chemistry, University of Pecs, Medical School, Pecs, Hungary; g Research Institute for Medicinal Plants and Herbs Ltd., Budakalász, Hungary; h Department of Molecular Biotechnology and Microbiology, Institute of Biotechnology, Faculty of Science and Technology, University of Debrecen, Debrecen, Hungary

**Keywords:** ELICE16INDURES®, Expression profiling, Defense mechanism, Soybean, *Glycine max*

## Abstract

Recently, climate change has had an increasing impact on the world. Innate defense mechanisms operating in plants - such as PAMP-triggered Immunity (PTI) - help to reduce the adverse effects caused by various abiotic and biotic stressors. In this study, the effects of ELICE16INDURES® plant conditioner for organic farming, developed by the Research Institute for Medicinal Plants and Herbs Ltd. Budakalász Hungary, were studied in a soybean population in Northern Hungary. The active compounds and ingredients of this product were selected in such a way as to facilitate the triggering of general plant immunity without the presence and harmful effects of pathogens, thereby strengthening the healthy plant population and preparing it for possible stress effects. In practice, treatments of this agent were applied at two different time points and two concentrations. The conditioning effect was well demonstrated by using agro-drone and ENDVI determination in the soybean field. The genetic background of healthier plants was investigated by NGS sequencing, and by the expression levels of genes encoding enzymes involved in the catalysis of metabolic pathways regulating PTI. The genome-wide transcriptional profiling resulted in 13 contigs related to PAMP-triggered immunity and activated as a result of the treatments. Further analyses showed 16 additional PTI-related contigs whose gene expression changed positively as a result of the treatments. The gene expression values of genes encoded in these contigs were determined by *in silico* mRNA quantification and validated by RT-qPCR. Both - relatively low and high treatments - showed an increase in gene expression of key genes involving AOC, IFS, MAPK4, MEKK, and GST. Transcriptomic results indicated that the biosyntheses of jasmonic acid (JA), salicylic acid (SA), phenylpropanoid, flavonoid, phytoalexin, and cellular detoxification processes were triggered in the appropriate molecular steps and suggested that plant immune reactions may be activated also artificially, and innate immunity can be enhanced with proper plant biostimulants.

## Graphical abstract

**Figure:**
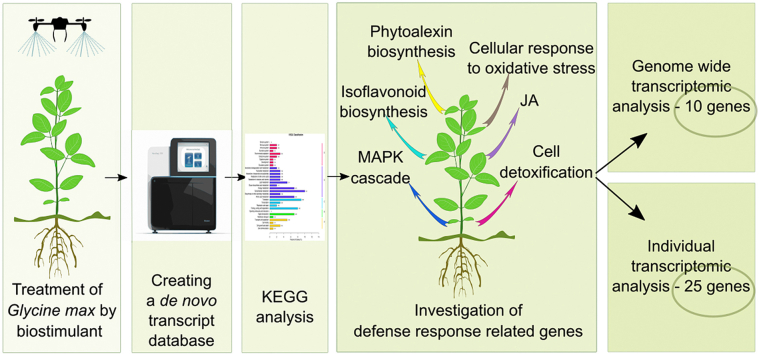

## 1 Introduction

The essence of the plant immune system is to be able to distinguish between their own and foreign substances and cells (viruses, bacteria, fungi, etc.) and to neutralize the extraneous substances after recognition. If the foreign intruder is not recognized and neutralized in time, a severe form of the disease may occur. Two types of defense systems can be distinguished in plants: (1) general (inherent) defense and (2) specific hypersensitive resistance (HR) [1]. Plants are characterized by the fact that each plant cell defends itself and carries out general and specific defense independently.

Plants react to infections by using their double-layered innate immunity systems. The first system recognizes and responds to molecules that are common to many classes of microbes, including non-pathogens, while the second one responds exclusively to the virulence factors of the pathogen, either directly or indirectly via their effects on host targets [2].

If a plant is able to recognize the foreign compounds produced by pathogens attacking it, active, inducible responses are created in the plants. Elicitors are special molecules produced by microbes that can trigger plant responses [3]. In plants, innate immune responses are mediated by special molecules. Examples of such molecules are pathogen-associated molecular patterns (PAMPs), which act on the surface of pathogens or in their intracellular organelles [4]. The ability of a pathogen-infected organism to recognize these patterns and mount an immune response results in broad-spectrum resistance to pathogens [5]. PAMP-triggered immunity may be the plant's first active response to the perception of the microbes [2,6]. In the first step of infection, PAMPs bind to recognition receptors and trigger an immune response that triggers gene expression in the infected plants, followed by the production of antimicrobial compounds [7]. PAMPs are compounds with conserved structures [8], and plant responses triggered by them are called innate or general immunity. PAMP-related defense responses and related biochemical pathways have long been widely studied [9,10,11,12].

According to current ideas, the inducible defense mechanisms in plants consist of a series of interconnected and complex stress responses, which can be triggered not only by microbial attack, but also by other stimuli [13]. New-generation plant conditioning compounds can provide an opportunity for such research, and the basic purpose of their development is to induce the plant's innate defense mechanisms and to stimulate the plant's health and immune system.

The exogenous application of plant-based biostimulants, such as herbal extracts with high immunochemical content, with different application techniques improves plant growth and yield, as well as the nutritional quality of the crop under stressful conditions. Medicinal plant extracts called phytochemicals may have antimicrobial activities on the development of plant pathogenic fungi [14,15]. Phytochemicals are biodegradable, nontoxic to plants, and have no residual effects [16]. They are composed mainly of secondary plant metabolites are organic compounds synthesized from primary metabolites (carbohydrates, proteins, lipids, and chlorophyll), and are considered biologically active compounds. Among these compounds, the followings possess the most important antimicrobial and antifungal activities: monoterpenes and diterpenes; steroids (derived from tetracyclic triterpenes); alkaloids; flavonoids; tannins and phenolic compounds. These substances may act effectively against fungal infections by affecting pathogens directly or indirectly by promoting plant systemic resistance [17].

The zigzag model of plant-microbe interactions is widely used in illustrating the nature of certain types of molecular interactions but it is not useful for quantification or prediction [18]. With the advent of faster, cheaper, and high-throughput “omics” methods, the ability to obtain large amounts of quantitative data is transforming and accelerating research in plant science and plant-microbial interactions [19,20].

The present study aimed to use transcriptomic methods to investigate the inducible defense processes in plants under non-pathogenic conditions.

For this purpose, the plant conditioner ELICE16INDURES® (RIMPH Ltd. Budakalász, Hungary) was used which is composed of herbal extracts containing the biologically active compounds mentioned above. This officially licensed product was developed based on an innovative green technology that improves plant vitality and resilience by stimulating their immune system (for its biologically active ingredients, see TableS 1). The advantage of this product is the recommended ultra-low volume (ULV) drone spraying application (20–60 g/ha, based on the authorization document) which increases the uniform spray pattern providing better homogeneity for the plants which leads to increases in yield levels [21].

In this study, ELICE16INDURES® was tested in two dosages, 20 g/ha (low dosage) and 240 g/ha (high dosage, approved by the authority), in a field experiment for a soybean population in Northwestern Hungary. Spraying was performed two times during the vegetation cycle. Although soybean is an economically important crop exposed to a wide range of biotic stressors, the defense responses induced by natural immunochemicals are less studied. The activation of biochemical pathways involved in plant immune responses was investigated - which are normally induced in response to biotic or abiotic stressors - after applying this plant conditioner. To exclude the effect of biotic stressors, only healthy, pathogen- and pest-free plants were analyzed. The here presented results discuss the dataset reported in our earlier paper [22].

Beneficial effects of ELICE16INDURES® treatments on plants (according to the information on the manufacturer's official websites https://gynki.hu/en/rimph-botanicals/products/and https://elicevakcina.blogspot.com/) - include better micronutrient incorporation and tissue strengthening, better qualities of the wax layer of the leaves and also for the fruits, formation of more compact crops, lower specific water consumption decreases and, consequently, improved drought tolerance of the plants, favorable changes in the contents and better functions of plant metabolism, increased saccharification (glycation), improvements in the quality of the carbohydrates, and more stable crop yields. Better shelf life and storability, as well as the increased transportability of off-season crop yields, and higher vitamin and mineral contents, resulted in better taste and aroma, and more balanced and more marketable crop yields of plant species with complex inflorescences and multiple seeds, and prevention and reduction of infections by pests (monilia, gray mold and sucking pests) are also listed as advantages.

## 2 Results and discussions

### 2.1 Remote sensing and sampling of field soybean population

**Fig. 1:**
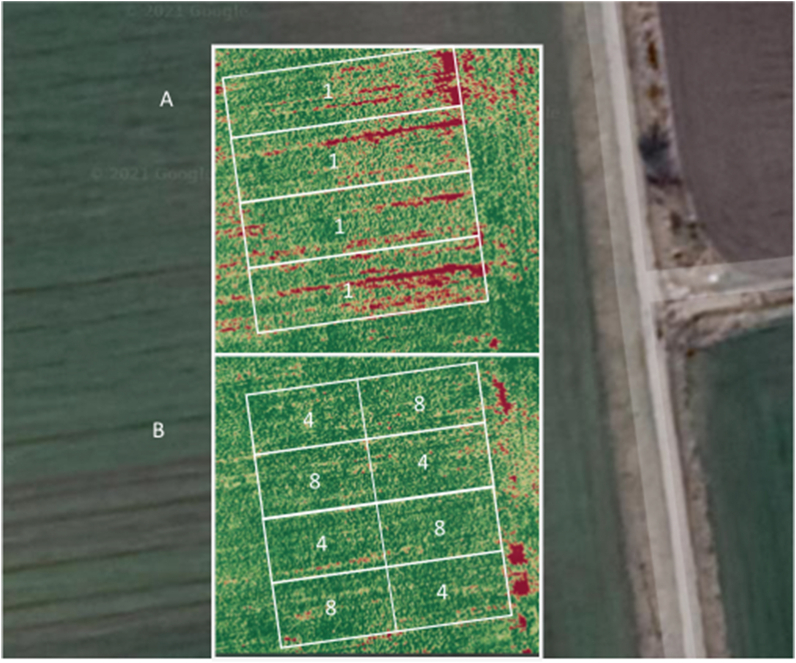
Soybean plots where data were collected by drone recording. “A” denotes untreated control (1), “B” marks treated plots where 4: sprayed with low dosage (20 g/ha), 8: sprayed with high dosage (240 g/ha).

After the treatments of the soybean plants in their early vegetative phase (May 25, 2020), data were recorded using a drone equipped with a near-infrared camera. With spectral analysis, the Enhanced Normalized Difference Vegetation Index (ENDVI) can provide information on the viability of field crops. By using drone recording the heterogeneity of the soybean population can be observed (Fig. 1a and b). The control (Fig. 1a) plant plots showed a lower vitality in contrast to sprayed (Fig. 1b). This data suggest that the ELICE16INDURES® plant conditioner can improve the vitality of plants even at a low dosage (20 g/ha). This higher vitality is due to better protection against various stressors, which was confirmed by measuring the mRNA levels of defense pathway genes. The recording was taken on July 08, 2020, during the critical seed-filling period of soybean phenophase, when plant vitality strongly influences the yield level.

Based on agro-drone and ENDVI data, samples for NGS sequencing were collected from control and treated plots. Sample collection was performed two times after the relatively low (20 g/ha) and high (240 g/ha) dosage treatments. The experimental design of treatments is reported in the earlier data article by Ref. [22] and is summarized in Fig. 6.

### 2.2 NGS sequencing and *de novo* transcriptome reconstruction

Six libraries were sequenced by Illumina NextSeq 550 technology. The resulting raw sequenced reads of each sample are summarized in Fig. 6. At the initial stage of the study, *de novo* assembly of the 6 × 9 M (approximately) cleaned combined read sets has resulted in 10, 492 total transcripts and 8308 total genes (supertranscripts). The percentage of GC was 51.71. The resulting reference transcriptome was analyzed at the gene level and deposited into the NCBI TSA database under the accession GJRQ00000000 (https://www.ncbi.nlm.nih.gov/nuccore/GJRQ00000000). Statistics of transcripts are detailed in the data article by Ref. [22]. Transcript abundancies were analyzed creating CountTable where the total mapped reads were presented for each transcript gene. The CountTable was deposited in the Mendeley database (https://data.mendeley.com/datasets/d2yypjh2hr/1).

### 2.3 Genome-wide transcriptomic analysis of differential gene expression

Using the NCBI TSA GJRQ00000000 transcript dataset, which represents a genome-wide transcriptome, pairwise differential gene expression analyses were performed. This investigation produced 292 differentially expressed contigs (TableS 2 https://data.mendeley.com/datasets/b2732cn4ts/1).

**Fig. 2:**
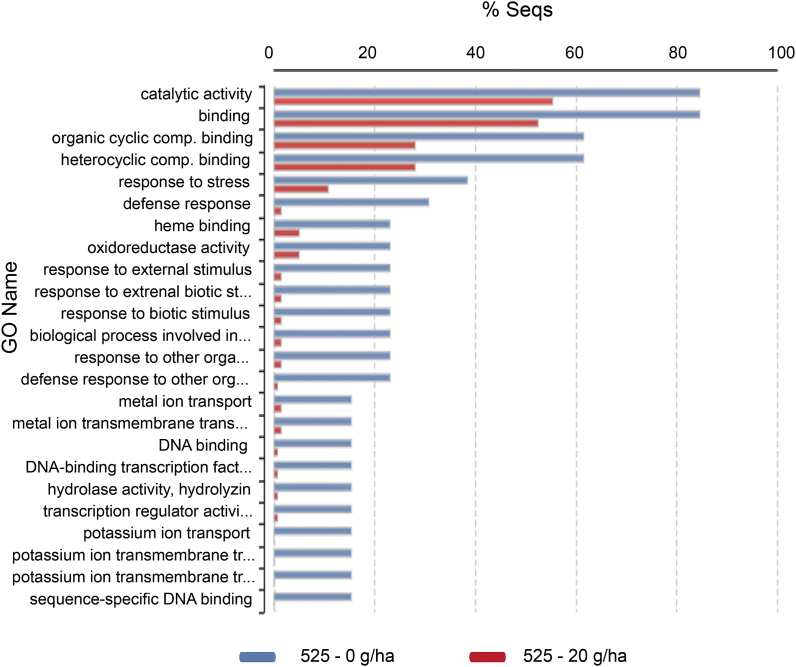
The biological role of upregulated, defense-related genes showing differentiated gene expression as a result of treatments. Bar chart representation of the biological roles of upregulated defense-related genes showing differential gene expression as a result of treatments. The figure shows the biological processes of the Gene Ontology (GO) term and summarizes the annotated genes of each group. Data reflect the expression differences between individual genes obtained by Fisher's Exact Test.

Further analysis of the 292 contigs was performed using the Fisher's Exact Test to determine which of them were up- or down-regulated (TableS 3 https://data.mendeley.com/datasets/b2732cn4ts/1) and then a heat map of 143 up- or down-regulated contigs was created (TableS 4). In this heat map, 13 upregulated contigs associated with the defense-pathways are marked in blue. The gene expression levels of the 13 contigs are represented in TableS5. These 13 upregulated contigs were blasted, annotated, and their roles in the biochemical processes were determined (Table 1). These contigs encoded 10 genes, which are discussed in detail below. Gene Ontology (GO) results of differential gene expression analysis are summarized in Fig. 2.

**Table 1:** Functional annotations of the upregulated contigs connected to the defense responses found in 525–4, 525–8, 710–4, and 710-8 samples (marked on the heat map TableS 4). References to genes with their roles in defense mechanisms and associated pathways are indicated. Notations: P: biological process, F: molecular function, C: cellular component.

Contig ID	Gene name and description	GO Names	Biochemical pathway	References
TRINITY_DN15551_c0_g1	7IOMT Isoflavone 7-O-methyltransferase	P:aromatic compound biosynthetic process; F:flavanone 4′-O-methyltransferase activity	isoflavonoid pathway	Akashi et al. 2003.
TRINITY_DN9412_c0_g1 TRINITY_DN1758_c0_g1	CYP82A3 cytochrome P450 82A3	P:defense response to other organism;	isoflavonoid pathway	Guttikonda et al. 2010.
TRINITY_DN4084_c0_g1	GST tau class glutathione S-transferase	P:glutathione metabolic process; F:glutathione transferase activity;	cell detoxification	Gullner et al. 2018.
TRINITY_DN12162_c0_g1 TRINITY_DN6147_c0_g1	WRKY48 probable WRKY transcription factor 48	P:regulation of transcription, DNA-templated; F:DNA-binding transcription factor activity;	SA pathway	Xing et al., 2008.
TRINITY_DN12168_c0_g1	AAA_ATPase AAA-ATPase ASD, mitochondrial-like isoform X2	F:ATP binding; F:ATP hydrolysis activity; C:integral component of membrane	SA pathway	Liu et al., 2020.
TRINITY_DN16956_c0_g1	WIP Wound-induced protein	P:defense response to bacterium; P:fefence response to fungus;	JA pathway	Takabatake et al., 2006.
TRINITY_DN17518_c0_g1 TRINITY_DN12230_c0_g1	MAPK Mitogen-activated protein kinase kinase kinase 17	F:protein serine/threonine kinase activity;	MAPK-cascade	Im et al., 2012.
TRINITY_DN9506_c0_g1	VH Vicianin hydrolase	P:carbohydrate metabolic process; F:hydrolase activity, hydrolyzing O-glycosyl compounds	Cyanogenesis pathway	Ahn et al., 2007.
TRINITY_DN12209_c0_g1	OsMAS Secoisolariciresinol dehydrogenase	F:momilactone-A synthase activity	phytoalexin biosynthesis	Atawong et al., 2002. Shimura et al., 2007.
TRINITY_DN8843_c0_g1	D6aH 3,9-dihydroxypterocarpan 6A-monooxygenase	P:defense response; F:3,9-dihydroxypterocarpan 6a-monooxygenase activity;	phytoalexin biosynthesis	Schopfer et al., 1998.

**Fig. 3:**
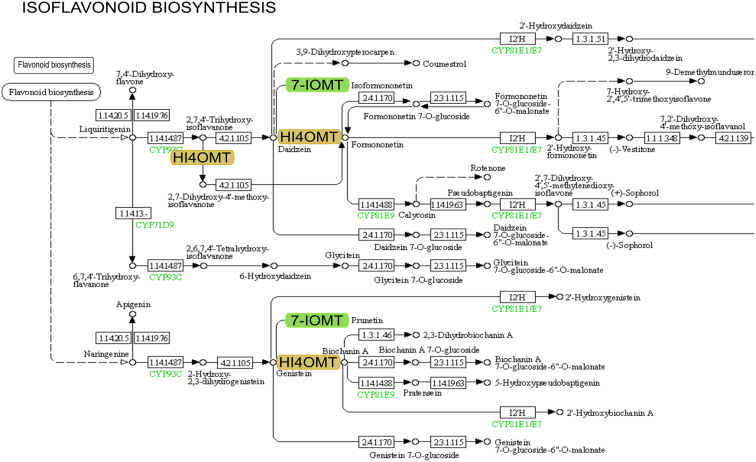
Based on the KEGG database, one of the genes was shown to be upregulated by the plant conditioning treatment, as the 7-IOMT enzyme, is located in the isoflavonoid biosynthesis pathway. The figure shows a part of the KEGG pathway of isoflavonoid biosynthesis (Abbreviations: 7-IOMT: isoflavone 7-O-methyltransferase, HI4OMT: 2-hydroxyisoflavanone 4′-O -methyltransferase).

The results of the genome-wide gene expression analysis showed that the treatments mainly induced defense-responsive genes. By analyzing the Kyoto Encyclopedia of Genes and Genomes (KEGG), we examined the participation of the genes listed in Table 1 in various biochemical processes. The results showed that the genes expressed after the treatments are involved in the following biochemical processes: isoflavonoid pathway (Fig. 3), cellular detoxification, JA pathway, SA pathway, cyanogenesis, phytoalexin synthesis, phenylpropanoid biosynthesis pathway, and MAPK cascade.

As a result of the applied plant biostimulator, the expression levels of the following genes were increased:

Isoflavone 7-O-methyltransferase (7IOMT) is a key enzyme in the isoflavonoid biosynthetic pathway (Fig. 3), which also plays a role in the biosynthesis of iso-formononetine and that of prunetin [23]. Isoflavones are abundant in soy, where they form an important class of compounds that mediate multiple plant-microbial interactions. Additional significant flavonoids such as pisatin synthesized in peas or the valuable soy phytoalexin glycerol [24] are produced in the isoflavonoid biosynthetic pathway, therefore, a more comprehensive study of the enzymes in the flavonoid biosynthetic pathway was performed.

Members of the cytochrome P450 (CYP, P450s) family of enzymes are involved in many biological processes [25,26], and the analysis also identified two contigs involved in the defense response as cytochrome P450 82A3. In addition, following the treatments, the glutathione S-transferase enzyme was also upregulated. This enzyme is involved in cellular detoxification processes [27].

The WRKY family of transcription factors (TFs) is the major group of TFs in higher plant species [28]. Various studies have confirmed the significant biological role of WRKY TFs in plant growth, and biotic and abiotic stress responses as they are also actively involved in metabolism, including carbohydrate synthesis, aging, and the production of secondary metabolites, as well as in plant responses to pathogen infection [29]. Based on other analyses, the transcription factor WRKY48 appeared to be upregulated and its roles in abiotic and biotic stress responses were demonstrated, among others, in *Arabidopsis* [30] that suggests an association between WRKY48 and the regulation of the SA pathway.

The gene of vicianin hydrolase enzyme was also found to be upregulated by the biostimulator treatments. Vicianin hydrolase is a cyanogen glucosidase that contributes to the breakdown of cyanogen glucosides in plant cells and their transformation into toxic hydrogen cyanide [31]. Cyanogenesis is a chemical defense response to cell- and tissue damage caused by herbivores and pathogens [32].

According to the literature, some members of the AAA-ATPase enzyme family are highly regulated in plants and can be associated with the activation of one of the known defensive metabolic pathways, the SA pathway [33]. Structurally, these proteins contain one or more conserved motifs, including the Walker A and Walker B motifs required for ATP binding and hydrolysis [34]. In plants, it has been reported that the AAA-ATPase genes from *Nicotiana tabacum* (NtAAA1) [35,36] and *Arabidopsis* (AtOM 66) [37] are negatively or positively affected in the SA signaling pathway and the hypersensitive response (HR) to pathogen infections.

In addition, it has been shown that a mutation-induced deficiency in the LMR/LRD6-6 (Os06g0130000) gene – which is also an AAA-ATPase – negatively affects HR and disease resistance in rice [38,39]. The ELICE16INDURES® treatment showed a significant increase in the activity of the AAA-ATPase enzyme-, which can be considered an immune response.

The CYP-dependent 3,9-dihydroxypterocarpane 6A-monooxygenase (D6aH) enzyme [40] was also upregulated by ELICE16INDURES® treatments. This enzyme is specifically involved in the biosynthesis of the inducible compound glyceollin, is a soybean phytoalexin [41,42]. The increased production of the enzyme may be paralleled by the upregulation of the above-mentioned CYP sequences and together they initiate phytoalexin synthesis as a defense mechanism in plant cells.

The results show that the activity of the secoisolariciresinol dehydrogenase enzyme was increased by ELICE16INDURES® treatments. The main function of this enzyme is the activation of momilactone A-synthase (OsMAS). Phytoalexin momilactone-A is a diterpenoid-type secondary metabolite involved in the plant's defense mechanism and produced in the phenylpropanoid biosynthetic pathway [43]. The formation of momilactone-A is induced by both abiotic and biotic stressors [44,45].

Wound-induced protein (WIP), which is reported to be closely related to the JA biosynthetic pathway [46], showed also an increased expression level. This protein helps to repair damages caused by wounds. In addition, two contigs indicate an increase in the expression level of MAPK17 which is a member of the MAPK enzyme family. The elements of the MAPK cascade have their primary roles in plant defense. This role of the MAPK cascade has been reported in several studies, including its key role in soybean defense responses [47].

To summarize, the differential gene expression analysis of treated field samples highlighted that JA, SA, phenylpropanoid, flavonoid, phytoalexin biosynthesis, and cellular detoxification pathways are involved in phenotypic reactions observed in drone recording and ENDVI. Therefore, the roles of these pathways were strengthened by further analysis. Additional genes of key enzymes of relevant biochemical processes were identified in field samples and individually analyzed by *in silico* mRNA level estimation.

### 2.4 Individual transcriptomic analysis of defense pathway genes

Based on the results obtained from the preliminary genome-wide gene expression analysis, a more comprehensive examination of additional 25 genes involving the stimulated biochemical pathways (JA, SA, phenylpropanoid, flavonoid, phytoalexin biosynthesis, and cellular detoxification pathways) was performed. These 25 genes with expression levels are presented in TableS5. These 25 genes were found in 23 contigs of combined transcriptome and showed that just after the first treatment of ELICE16INDURES® 966,199 of the 9,919,253 RNA-Seq reads could be aligned to the investigated genes in the samples treated with the lower dosage.

#### 2.4.1 The first treatment by a low dosage of ELICE16INDURES®

**Fig. 4:**
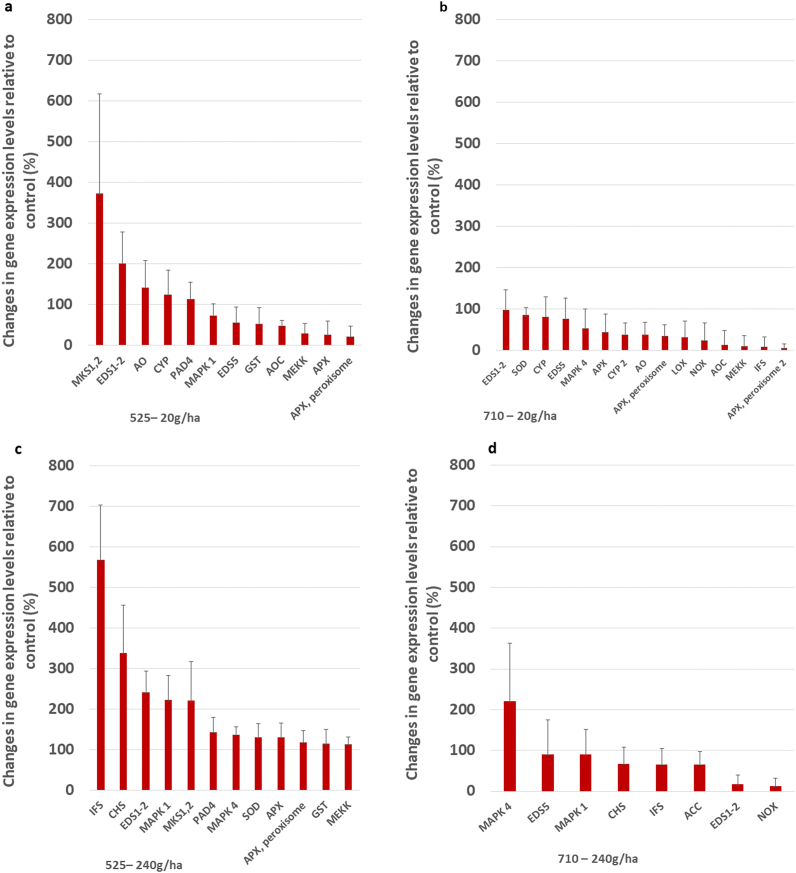
Expression analyses of GOIs *in silico*. Genes showing upregulation and related to defense-responses in the samples collected at two sampling times of the two treatments. Mean values are plotted and the standard deviation of the four biological replicates is shown. Data show increases in gene expression values in percentages compared to the control conditions.

In this study, the expression level of the MAPK cascade enzyme genes (MAPK1, MEKK) strongly increased after the first intervention due to the lower dose of 525–4 treatment (Fig. 4a). Elevated levels of the MAPK cascade enzymes are the first demonstrable signs of the immunostimulating effect of the tested substance. The first step in protecting plants against stress is recognition and then signal transmission through the signal transduction system, which includes, for example, the MAPK pathway [47,48]. When an infection is detected, the receptors on the surface of the plant cell immediately send a message to the cell nucleus about the presence of a virulent microorganism. This process occurs through the MAPK cascade pathways. MAPK can be activated by both abiotic and biotic stressors. This system is highly conserved in all eukaryotic cells. Several studies have investigated the role of the MAPK cascade in plants [49] include parsley [13]; *Arabidopsis* and [47] soybean. Various abiotic stressors induce many cascades [50,51]. According to current research, MAPKs play a significant role in defense against pathogens in *Arabidopsis* [52,53], tobacco [54], tomato [55], parsley [56], brassica [57] and rice [58].

Our results suggested that MAPK signaling may be induced by exogenous phytochemicals or phytohormones if they contact the plant surface. In the investigated agent, cell-identical liposomal encapsulation helps the better uptake and delivery of the active compounds of plant extracts. Presumably, the enhanced MAPK signaling may be one of the plant cell responses to increased phytohormones deriving from the investigated agent. This reaction may be similar to the herbivore and pathogen defense regulation, such mechanism is connected to jasmonic acid, ethylene, and salicylic acid, and MAPKs are also required for transcriptional activation of defense-related genes and accumulation of defensive metabolites [59].

The level of the enzyme amine oxidase (AO), - which catalyzes the synthesis of locally produced H_2_O_2_ in different cellular organs after infection, - was also found to be increased in this experiment. In recent research, it has been proven that the enzymes of the AO protein family play a role in the synthesis of H_2_O_2_ [60], which is formed separately in different cell organelles. It is also hypothesized that H_2_O_2_ is likely to play a key role in preventing biotic stressor-induced infections [61]. As a result of the previous effects, the gene expression level of the ascorbate peroxidase (APX) enzyme also increased, which can also indirectly explain the quantitative increase in H_2_O_2_ production [62]. In plants, APXs can neutralize the accumulated H_2_O_2_ by converting it to water [63]. Egbichi et al. reports on elevated H_2_O_2_ levels associated with increased APX levels in soybean following the application of an external stressor as an abiotic stressor [64]. The increased mRNA levels of AO, APX, and 7IOMT (the key enzyme involved in the biosynthesis of flavonoids - as well-known antioxidants, see above) in the treated samples suggested a key role of the enhanced antioxidant enzyme system involved in defense-response triggered by the investigated biostimulator.

Treatments with ELICE16INDURES® also had a positive effect on increasing the level of the CYP enzyme, thereby catalyzing the plant's phytoalexin production too [65]. Gene expression changes of the CYP enzyme family and their effects were also investigated in soybean [25]. The CYP superfamily is the largest family of enzymatic proteins found in all living organisms. P450s play a role in protecting plants from various abiotic and biotic stresses. They are also involved in hormone metabolism and phytoalexin biosynthesis [66,67]. Phytoalexins are broad-spectrum inhibitors that are used by plants as a rapid defense response against a variety of stressors. According to our hypothesis, the elevated level of the CYP enzyme may be associated with an increase in the antioxidant capacity of plants and the resulting stress tolerance. This assumption seems to be supported by the fact that as a result of this plant conditioning treatment, an increase in the expression levels of Enhanced Disease Susceptibility 1-2 and its co-regulator Phytoalexin Deficient4 enzymes (EDS1-2 and PAD4, respectively) were also observed after this treatment, which contributes to increased phytoalexin production. Primary plant defense responses also include the synthesis of pathogen-related (PR) proteins and phytoalexins. According to Wang et al. EDS1-2 and PAD4 are well-known regulators of both basal and resistance (R) gene-mediated resistance [68]. Two EDS1-like (GmEDS1a/GmEDS1b) and one PAD4-like (GmPAD4) proteins have been recognized in soybean, which play a significant role in resistance signaling. According to the study, the expression of EDS1 and PAD4 genes was increased by biotic stressors [68].

The regulatory role of the EDS1-2/PAD4 genes in the control of the SA pathway has already been described in *Arabidopsis* plants. Recent research has revealed that these two enzymes may work in parallel but independently in the regulation of the SA pathway [69]. Further transcriptomic research showed another rame of EDS1/PAD4-regulated pathways that were independent of SA and were involved in basal and TNL immunity [70,71,72,73,74]. In cases where the SA pathway is inadequate (e.g following a pathogen attack), the EDS1-2/PAD4 pathway can be activated contributing to resistance and plant protection as a helping mechanism. This process may strengthen the plant's inherent immunity [69]. The changes in the regulation of the EDS1-2/PAD4 genes in our results reinforce the assumption that ELICE16INDURES® treatment affected the SA-regulated defense responses of investigated plants. The Enhanced Disease Susceptibility 5 (EDS5) gene is also closely related to the plant defense system, resulting in enhanced disease resistance [75]. The EDS5 gene expression level is particularly low in healthy plants, but increases significantly in parallel with the EDS1-2/PAD4 genes upon infection [76]. Although EDS5 enzyme levels are extremely low in healthy plants, the ELICE16INDURES® biostimulant in this experiment successfully induced higher expression of this gene, thereby enhancing the disease resistance of plants, which result can be considered a novel one in studying biostimulants. The EDS5 protein also elicits immunity induced by basal resistance or PAMPs [68,76]. The role of EDS5 in the ICS branch of SA biosynthesis pathway has so far only been characterized in *Brassicaceae* plants [77], and this pathway of SA biosynthesis may not be complete or functional in other plant families. Therefore, changes in the expression of the EDS5 enzyme and its associated biochemical pathways require further research in other plants, considering the obtained results in soybean plants.

As a result of the plant conditioner treatments applied here, the AOC enzyme mRNA level also showed a significant increase. The initial steps of the JA biosynthetic pathway include the sequential conversion of α-linolenic acid (LnA) into OPDA by lipoxygenase (LOX), allene-oxide-synthase (AOS), and AOC, which operate in the chloroplasts [78,79,80,81].

Overexpression of these genes indicates that the JA biosynthesis pathway was induced by the treatments. While SA regulates protection against biotrophic pathogens, JA and methyl-jasmonate (MeJA) primarily regulate immunity to necrotrophic pathogens and pests [82,83].

The former finding is also supported by a significant increase in the expression level of the enzyme methyl ketone synthase (MKS-1,2) in this experiment. MKS enzymes are induced by MEKK enzymes the latter are involved in MeJA synthesis. This experiment indirectly demonstrated that the ELICE16INDURES® may stimulate MeJA synthesis.

An additional general cellular defense response is a sudden increase in the levels of plant GSTs. The expression level of the GST enzyme, which is involved in general cell detoxification processes was also increased in the first ELICE16INDURES® experiment. The GSTs are encoded by large gene families. These genes are present almost everywhere and their expression can be triggered by various stresses. In plants, these enzymes are mostly induced in the early stages of bacterial and fungal infections, which is due to the detoxification effect, however, recent research has also revealed new relationships [84,85], including the down/up-regulation of GSTs can affect the rate of pathogen reproduction and the severity of the caused disease [27]. It is well documented that GST enzymes in higher plants may be induced by a variety of fungal pathogens, xenobiotics and elicitors, wounding, and phytohormones [86,87,88,89,90]. In this study, the increased GST mRNA levels suggested that the investigated treatments may trigger the plant detoxification processes by eliminating damaged plant cell components, presumably.

#### 2.4.2 The second treatment by a low dosage of ELICE16INDURES®

In the population treated with a lower dosage of this plant conditioner 786,171 of 10, 059, 111 RNA reads of the second at the second sampling time were assembled to 25 gene sequences which produced 23 contigs.

The treatment with a lower dosage but at a later time increased the gene expression of some enzymes of the MAPK cascade in the soy plants (Fig. 4b).

In addition, the expression levels of NADPH oxidase (NOX) and superoxide-dismutase (SOD) enzymes were found also to be increased. NOX uses oxygen to produce superoxide anions (O2^•^) while SOD catalyzes the conversion of the superoxide radical to H_2_O_2_ so the enhanced defense function of the plant cell wall is also manifested here.

Plasma membrane NOXs are key producers of the Reactive Oxygen System (ROS) and play important roles in the regulation of plant-pathogen interactions. Zhang, Zhao [91] identified 17 NOX (GmNOX) genes in the soybean (*Glycine max*) genome. Oxidative damage caused by ROSs may occur to various stresses in plants among which next to the biotic pathogens, the most significant abiotic stresses are toxic metals, high temperatures, drought, and salinity. To scavenge ROS and avoid oxidative damage in soybean SOD is an enzyme that alternately catalyzes the partitioning of the superoxide (O^2• -^) radical produced as a by-product of oxygen metabolism into molecular oxygen (O_2_) and H_2_O_2_ [92,93].

The level of the AO enzyme - which catalyzes the synthesis of H_2_O_2_ produced by cellular organs – was also increased by this treatment. Besides NOXs, apoplast AOs are enzymatic sources of ROS production [94,95,96,97]. These H_2_O_2_-triggered enzymes have been shown to regulate the initiation of lignification and programmed cell death as the result of biotic and abiotic stresses [94,[98], [99], [100], [101], [102]]. The level of APXs, which are responsible for neutralizing the needless redundant H_2_O_2_ was also increased. From the above findings, it is suggested that the ELICE16INDURES® treatments may act as weak stressors (eustressor) on the plants, thereby exerting their stimulating effects. In the beginning, they increase the production of the activated oxygen forms in the plants, then simultaneously activate the production of those enzymes that neutralize them.

Similar to the first sampling time, gene expression of CYP, EDS1-2 and EDS5 enzymes enhanced, increasing the induction of phytoalexin production. In addition, the second time treated samples, the level of IFS also enhanced. Lozovaya et al. transformed soybean roots with gene sequences derived from the IFS enzyme. The method increased the plant's phytoalexin (glycerol) production and leads to resistance against pathogenic fungi [103]. Lygin et al. (2013) showed that the rapid accumulation of glyceollin during infection promotes the defense response of soybean (Lygin, Zernova [104]). The accumulation of IFS, which plays a role in the synthesis of phytoalexins, seems to support our earlier hypothesis for the CYP enzyme, that the stress tolerance of the plants can be increased by the ELICE16INDURES® biostimulator treatments - presumably through the mediation of phytoalexins that are formed.

In addition, after this plant conditioning treatment, the level of the LOX enzyme - involved in the production of oxylipins (such as JA) - also increased. JA and its derivatives are known to play a significant role in the development of defense responses [83]. A positive change in the gene expression level of LOX, which plays an important role in the biosynthesis of JA, may be related to the development of a possible defense response. The JA biosynthesis pathway can usually be associated with pests, but these were not present in our experiment. From this, can be concluded that the active ingredients of the plant conditioner when in contact or penetrating the cells are capable of increasing JA biosynthesis and thus resulting in immunity.

#### 2.4.3 The first treatment by a high dosage of ELICE16INDURES®

In the population treated with the higher dosage of plant conditioner, after the first treatment 722,540 of 9,892,689 RNA reads were assembled to 23 contigs involving 25 gene sequences.

In the population treated with a higher dosage of this substance, the expression levels of the MAP cascade-related MAPK1, MAPK4, MEKK, and MKS 1,2 enzymes increased at the first time of the treatment (Fig. 4c). Each of these enzymes is responsible for the development of the defense responses. The gene expression level of SOD and APX enzymes, which play a role in the thickening of the cell wall, increased significantly; while the levels of chalcone synthase (CHS), IFS and EDS1-2/PAD4 enzymes supporting phytoalexin synthesis also enhanced. In addition, the gene expression level of the GST enzyme responsible for cell detoxification processes also improved.

#### 2.4.4 The second treatment by a high dosage of ELICE16INDURES®

In the population treated with a higher dosage of plant conditioner after the second treatment 566,317 of 8,795,753 RNA reads were assembled to 25 gene sequences producing 23 contigs.

A higher dose of plant conditioner secondly (Fig. 4d) induced a MAPK cascade (MAPK1, MAPK4) and increased NOX activity. The latter promotes the formation of reactive oxygen species that play a role in the thickening of the cell wall. Furthermore, the level of the acetyl-CoA carboxylase (ACC) enzyme, which is the first enzyme in the process that initiates the production of phytoalexins, has been improved [105]. The enzyme ACC converts acetyl-CoA to malonyl-CoA; then CHS promotes the formation of the flavonoid structure, and also enhance the gene expression level of the IFS enzymes that catalyze the biosynthesis of isoflavones. As a result of this plant conditioning treatment, the gene expression level of both enzymes (CHS and IFS) increased significantly. The level of EDS1-2 and EDS5 enzymes – which are responsible for the cell-protective phytoalexin synthesis – also showed an increase in this experiment. The increase in expression of these genes confirms what was previously detected after ELICE16INDURES® treatments, that the plant conditioner activated several enzyme genes involved in the biochemical pathway of phytoalexin synthesis.

To summarize, we investigated genes of 25 enzymes having a key role in the defense-mechanisms involving JA, SA, phenylpropanoid, flavonoid, phytoalexin biosynthesis, and cellular detoxification pathways. Among these, 16 genes in total were found to be overexpressed in the samples collected after the first and second treatments of low and high dosages. The overexpressed genes are summarized in Table 2.

**Table 2:** Annotation of the 16 upregulated genes found in investigated pathways. During the annotation process, GO name, enzyme name, and enzyme code were identified for the investigated sequences (see contig ID).

Contig ID	Gene name and description	GO Names	Enzyme Name and code	Biochemical pathway
TRINITY_DN16_c0_g1	GST glutathione-s-transferase 3	P:glutathione metabolic process; F:glutathione transferase activity;	glutathione-s-transferase 3 EC:2.5.1.18	cell detoxification
TRINITY_DN993_c0_g1	APX l-ascorbate peroxidase 3, peroxisomal	P:response to reactive oxygen species; P:cellular response to oxidative stress; P:hydrogen peroxide catabolic process; P:cellular oxidant detoxification; F:l-ascorbate peroxidase activity;	l-ascorbate peroxidase EC:1.11.1.11	cell detoxification
TRINITY_DN10910_c0_g1	SOD superoxide-dismutase 1	C:membrane; C:integral component of membrane	superoxide-dismutase 1 EC:1.15.1.1	cell detoxification
TRINITY_DN13696_c0_g1	AOC allene-oxide cyclase	P:regulation of transcription, DNA-templated; P:protein transport;	allene-oxyde-cyclase EC:5.3.99.6	JA biosynthesis
TRINITY_DN4965_c0_g1	MAPK4 mitogen-activated protein kinase 4	P:signal transduction; P:transmembrane transport; F:phosphorelay sensor kinase activity;	Mitogen-activated protein kinase 4 EC:2.7.11.24	MAPK cascade
TRINITY_DN2507_c0_g1	MAPK3 mitogen-activated protein kinase 3	P:MAPK cascade; P:response to oxidative stress; P:camalexin biosynthetic process; P:defense response to bacterium P:defense response to fungus; P:priming of cellular response to stress;	Mitogen-activated protein kinase EC:2.7.11.24	MAPK cascade
TRINITY_DN4132_c0_g1	MAPKKK1-like isoform X1	P:MAPK cascade; F:MAP kinase kinase kinase activity;	Mitogen-activated protein kinase kinase kinase EC:2.7.11.25	MAPK cascade
TRINITY_DN10501_c0_g1	MKS1 methylketone-synthase 1 b	P:phosphorylation; F:protein kinase activity; F:kinase activity;	methylketone-synthase 1 Gene ID: 101,265,685	JA biosynthesis
TRINITY_DN1751_c0_g1	PAD4 lipase-like Phytoalexin Deficient4	P:lipid metabolic process; F:hydrolase activity; C:nucleus	phytoalexin deficient 4 EC:2.3.1.-	phytoalexin biosynthesis
TRINITY_DN6664_c0_g1	EDS1-2 Enhanced disease susceptibility 1-2	P:lipid metabolic process; P:defense response	hydrolases EC:3	phytoalexin biosynthesis
TRINITY_DN18235_c0_g1	EDS5 Enhanced disease susceptibility 5	P:xenobiotic transport; P:transmembrane transport; F:antiporter activity;	translocases EC:7	phytoalexin biosynthesis
TRINITY_DN14164_c0_g1	NOX respiratory burst oxidase homolog protein C	P:defense response to virus; P:cellular oxidant detoxification; F:peroxidase activity; F:NAD(P)H oxidase H2O2-forming activity;	NAD(P)H oxidase (H (2)O (2)-forming); Acting on a peroxide as acceptor EC:1.6.3.1; EC:1.11.1	oxidative stress
TRINITY_DN17316_c0_g1	IFS isoflavone synthase 2	P:isoflavonoid biosynthetic process; F:2-hydroxyisoflavanone synthase activity;	EC:1.14.14.87; EC:1.14.13.86	isoflavonoid pathway
TRINITY_DN603_c0_g1	ACC-ase acetyl-CoA carboxylase 1	P:malonyl-CoA biosynthetic process; F:acetyl-CoA carboxylase activity;	Acetyl-CoA carboxylase EC:6.4.1.2	isoflavonoid pathway
TRINITY_DN18316_c0_g1	CYP cytochrome P450 83B1	F:monooxygenase activity; F:oxidoreductase activity,	Acting on paired donors, with incorporation or reduction of molecular oxygen EC:1.14	isoflavonoid pathway
TRINITY_DN3368_c0_g1	CHS chalcone synthase	P:flavonoid biosynthetic process; F:naringenin-chalcone synthase activity	Chalcone synthase EC:2.3.1.74	isoflavonoid pathway

The change in the expression levels of the other investigated enzymes did not show any significant difference as a result of the treatments recorded on different sampling dates.

### 2.5 RT-qPCR validation of genes of interest (GOIs)

**Fig. 5:**
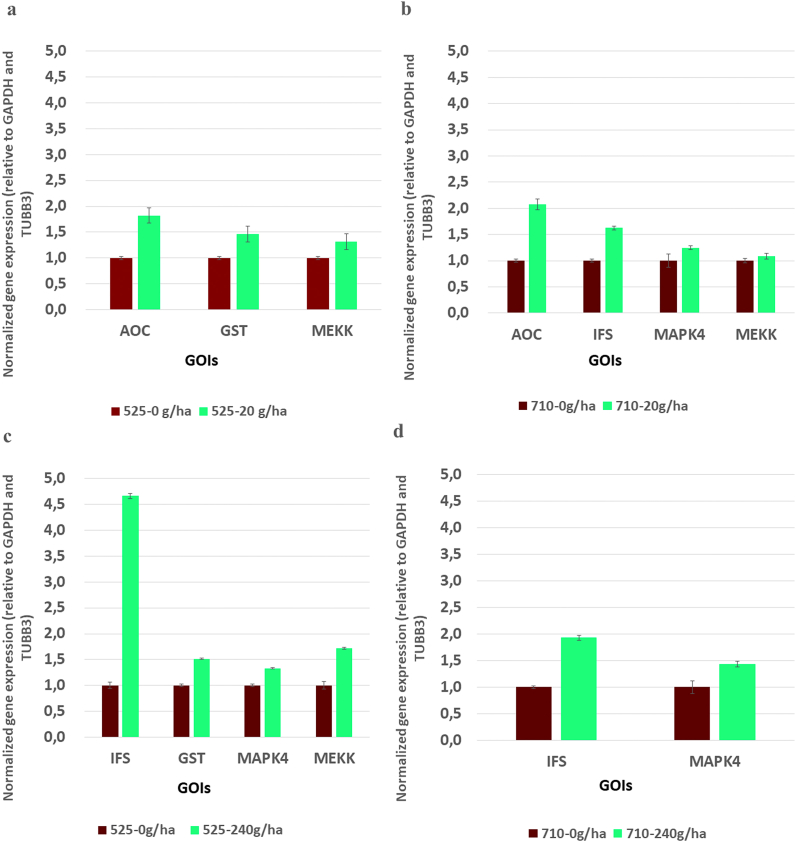
RT-qPCR amplification of GOIs. Relative gene expression was determined for GAPDH and TUBB3 reference genes. Values are mean ± SEM (n = 4). p < 0.05 vs. control, p < 0.05 vs. treated group.

To support the *in silico* results, RT-qPCR validation of 5 selected GOIs from the samples 525–1, 525–4, 525–8, 710–1, 710–4, and 710–8 (Fig. 5) was performed (raw data of RT-qPCR quantification can be found at https://data.mendeley.com/datasets/3nk3x3n64v/1). Based on the above-detailed results, the following genes were selected for validation: AOC, GST, MEKK, IFS, and MAPK4. All the genes included in the RT-qPCR assays showed similarly elevated gene expression as in the *in silico* analysis of the respective treatments. These data indicate that the increased expression levels of these genes were maintained during the maturation of soybean plants, except for GST. This might be explained by that the GST enzyme plays a role in the detoxification processes of cells, and after these processes are completed, the function of the enzyme decreases to its basic level. These results may support the assumption that the cellular immune response may be triggered by using biostimulator treatments without abiotic or biotic stress suffering.

## 3 Conclusions

Recently, the role of alternative plant conditioners in sustainable plant protection is receiving increased attention. Many products are on the market which strive to combat different abiotic or biotic stressors. However, the future of plant protection technologies lies in the natural strengthening and stimulation of the plant immune system. The green technology-based ELICE16INDURES® containing 11 botanical extracts can be successfully applied in organic farms due to its benefits on the vitality of plants. Healthier crop cultures can be well monitored, as presented here by agro-drone recordings. To better understand the effects of the applied biostimulator agent, gene expression profiling was performed twice during the life cycle of the soybean plants. These genome-wide data revealed the inducibility of some immune response genes. Based on these data, some of the genes involved in JA, SA, isoflavonoid, and phytoalexin biosynthesis, and those of the MAPK cascade, cellular detoxification, and responses to oxidative stress were activated by the biostimulator applied here. We consider it outstanding results of the upregulation of genes involved in flavonoid biosynthetic pathways like CYP, IFS, ACC, CHS, EDS1-2, EDS5 affecting the production of flavonoids, antioxidants, and phytoalexins in healthier soybean plants of ELICE16INDURES® treated plots. The *in silico* predicted stress responses were confirmed by the higher expression of selected genes in RT-qPCR validation experiment. The results presented here suggest that the plant immune system can be induced by the applied immunochemical substances leading to phenotypical alterations such as healthier plants and contributing to the development of green plant protection technologies.

## 4 Materials and methods

### 4.1 Plant materials

**Fig. 6:**
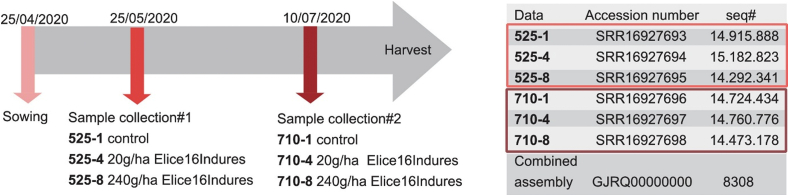
Representation of the experimental design. Timeline of samples collected in field-grown soybean. Numbers denote samples as follows: 525 means samples, collected on 25, May 2020; 710 means those, collected on 10 July 2020; ELICE16INDURES® treatments of 1–0 g/ha (control); 4–20 g/ha and 8–240 g/ha. Sample marks were indicated with NCBI data deposition numbers. Combined assembly means *de novo* Glycine max transcriptome dataset [22].

*Glycine* max cv. ES Director (maturity group 00, Euralis, 2017) plants were cultivated in field conditions at Tata in Hungary. Plants were sprayed by TTAM4E drone using low and high (20 g/ha and 240 g/ha) dosages of ELICE16INDURES® in four repetitions block systems (Fig. 1.) Samples were taken from both untreated and treated plots, two days after spraying two times (25 May and 10 July), in 2020. The sample collection and storage conditions were published by Ref. [106]. The four replicates of each sample were pooled and sequenced by Xenovea Ltd. (Szeged, Hungary). The samples of different collection times and dosages are summarized in Fig. 6.

### 4.2 Remote sensing

To monitor the health of the plants in field populations of soybean, a DJI-phantom 4 agro-drone equipped with a near-infrared camera was employed. Drone monitoring was performed on July 08, 2020. Drone photos (GeoTiff; GPS coordinates: 47 deg 37′58.30″N, 18deg15′54.36″E) were processed with Dronedeploy (https://www.dronedeploy.com/). Since healthy plants reflect both visible green and near-infrared (NIR) lights, the ENDVI was used as the indicator of soybean plant health. ENDVI comprises the comparison of green and blue lights in addition to the NIR and red ones to give a sensitive result (Equation (1)).

(1) $$ENDVI=\frac{\left(\left(NIR+Green\right)-\left(2xBlue\right)\right)}{\left(\left(NIR+Green\right)+\left(2xBlue\right)\right)}$$

ENDVI combines the NIR and Green channels together for the reflective channel. The Blue channel is multiplied by two to compensate for the combination of the NIR and Green channels.

### 4.3 NGS library preparation and sequencing

NGS libraries were constructed by using QuantSeq 3'mRNA-Seq Library Prep Kit FWD for Illumina (Lexogen GmbH, Wien, 510 Austria) according to the manufacturer's protocol. Diluted samples (dilution to 1.8 pM) were sequenced using NextSeq 500/550 High Output v2 Kit (75-cycle) on the NextSeq 550 platform (Illumina, San Diego, CA, USA) to produce 1 × 86 bp single-end reads. Using QuantSeq the 3′ end of poly(A) RNA may be pinpointed by obtaining accurate information about the 3′ UTR.

### 4.4 Pre-processing and assembly

Reads were pre-processed using Trimmomatic software [107]. This software allows to removal of the adapters and contaminating sequences. Transcriptome assembly with cleaned and combined read sets was performed by using Trinity and Bowtie2 programs [108,109].

### 4.5 Functional annotation

Functional annotation and Gene Ontology (GO) analyses were carried out using OmicsBox.BioBam program package [110], as follows: sequences were blasted by NCBI non-redundant Viridiplantae database (downloaded in 2022) applying blastx configuration. To retrieve gene ontology (GO) terms associated with the first 15 hits obtained by the Blast search, GO mapping and annotation were performed. GO annotation was used to determine the molecular function, intracellular location, and role of the genes in the biochemical processes.

### 4.6 Estimating transcript abundances by read mapping

To estimate transcript abundances each sample reads were aligned to the *de novo* transcriptome assembled from the combined read sets. The number of reads for each feature is presented in the CountTable (see Supplementary 2). This process was performed by using the HTseq package [111] and Bowtie2.

### 4.7 Pairwise differential expression analysis

Contigs from *de novo* transcript were used to create the CountTable (https://data.mendeley.com/datasets/d2yypjh2hr/1). Differentiated gene expressions (TableS 2 https://data.mendeley.com/datasets/b2732cn4ts/1) were calculated based on the CountTable, that is, with how much abundance can the reads of the different samples fit for a particular gene). For these calculations pairwise differential expression analysis without replicates was applied by NOISeq program [112] were used. NOISeq is a nonparametric approach for the identification for differentially expressed genes from RNA-Seq count data. It creates a null or noise distribution of count changes by contrasting fold-change differences (M) and absolute expression differences (D) for all the genes in samples within the same condition. This reference distribution is then used to assess whether the M and D values computed between two conditions for a given gene are likely to be part of the noise or represent a true differential expression. The replicate simulation relies on the assumption that read counts follow a multinomial distribution, where probabilities for each class (feature) in the multinomial distribution are the probability of a read to map to that feature. These mapping probabilities are approximated by using counts in the only sample of the corresponding experimental conditions. Given the sequencing depth (total amount of reads) of the unique available sample, the size of the simulated is a percentage of this sequencing depth, allowing a small variability.

### 4.8 Enrichment analysis (Fisher's exact test)

Gene Set Enrichment Analysis (GSEA) was used to determine those genes whose expression showed a statistically significant difference between two biological samples (https://data.mendeley.com/datasets/b2732cn4ts/1). Fisher's Exact Test [113] was used to find GO terms that are up-and downregulated in a set of genes with respect to the reference (untreated) group.

### 4.9 Determination of sequences of defense pathway genes

After screening the entire transcript dataset those pathways, which are stimulated by the ELICE16INDURES® (TableS 4) were determined. These pathways were further analyzed. Gene sequences of enzymes involved in the metabolic processes were collected from known related species (*Glycine* max and *Glycine soja*) using the NCBI public database (https://www.ncbi.nlm.nih. gov/nuccore). In the next step, expressed contigs were retrieved from the *de novo* transcriptome for control and treated soybean plants by using local Blast+ [114].

### 4.10 Pathway analysis

In order to get a strict overview of the biochemical pathways triggered by these immunostimulant treatments, pathway analysis was performed by aligning sequences to the KEGG database. This was carried out to summarize the information and to interpret the results with a greater precision [115].

### 4.11 Expression analyses of GOIs *in silico*

Individually digital expression analysis of genes of interest (GOIs) were performed as follows: short reads of transcripts obtained from the control and those from the treated plants were aligned to the reference sequences using Bowtie2, and these data were further analyzed. First, individual RPM (Reads Per Million mapped read) values were calculated for each gene (Equation (2)) in four biological replicates, and these values were then presented in figures for each treatment and time point (Fig. 4).

(2) $$RPM=\frac{mapped\;reads\;to\;a\;gene\;x\;10^{6}}{total\;mapped\;reads}$$

In some special RNA-seq protocols, especially in the shallow RNA-seq methods, reads are generated only from one end of the RNA molecule regardless of length. The RPM gene expression index does not take into account the length of the transcript and therefore more accurately reflects the actual number of transcripts. We used the RPM values to determine the individual expression levels of the enzyme sequences operating in the given treatment at a proper time, i.e. the degree of their real expression. From the RPM values, the individual expression levels of the enzyme sequences triggered by the treatments applied at a particular time were obtained [116].

### 4.12 Expression analysis of GOIs with RT-qPCR amplification

RT-qPCR experiments of selected GOIs such as AOC, GST, IFS, MAPK4, MEKK were performed as described earlier [117]. Primers (TableS 6) were designed with Primer 3 [118] based on *in silico* sequences of GEx libraries. Gene expression analysis was established based on three technical and biological replicates and normalized using the reference genes of glyceraldehyde-3-phosphate dehydrogenase (GAPDH) and β-tubulin (TUBB3). Statistical analysis was performed by analysis of variance, and all of the data were expressed as the mean ± SEM. The homogeneity of the biological replicates was tested by F-test (Levene's test). There were no significant differences among these groups. Comparisons among the treatment samples were made by one-way ANOVA with a post hoc correction (SPSS for Windows, version 21.0). The Student's t-test was used to compare the mean values of groups. A value of p < 0.05 was considered statistically significant.

## Author contribution statement

Kincső Decsi: Analyzed and interpreted the data; Wrote the paper. Barbara Kutasy, Géza Hegedűs, Zoltán Péter Alföldi: Contributed reagents, materials, Analysis tools or data. Nikoletta Kálmán: Contributed reagents, materials, analysis tools or data. Ágnes Nagy: Conceived and designed the experiments; performed the experiments. Eszter Andrea Virág: Conceived and designed the experiments; performed the experiments; analyzed and interpreted the data.

## Funding statement

Eszter Andrea Virág was supported by 10.13039/501100015269Hungarian Government [KFI_16-1-2017-0457 - Development and production of a plant-based pesticide-plant conditioner for use in organic farming - project], 10.13039/501100012550Nemzeti Kutatási, Fejlesztési és Innovaciós Alap [KFI_16-1-2017-0457 - Development and production of a plant-based pesticide-plant conditioner for use in organic farming - project].

## Data availability statement

Data associated with this study has been deposited at PRJNA778970, SRA submission SUB10637174. TSA (Transcriptome Shotgun Assembly) data was deposited in the NCBI under the accession PRJNA778970, TSA temporary submission SUB10815164M.

Count table: https://data.mendeley.com/datasets/d2yypjh2hr/1.

DEG table, up- and downregulated contigs table: https://data.mendeley.com/datasets/b2732cn4ts/1.

Results of RT-qPCR quantification data https://data.mendeley.com/datasets/3nk3x3n64v/1.

## Declaration of competing interest

The authors declare no competing interests.
