# Corrigendum to “Natural immunity stimulation using ELICE16INDURES® plant conditioner in field culture of soybean” [Heliyon 9 (1), (January 2023) Article e12907]

**DOI:** 10.1016/j.heliyon.2023.e13954

**Published:** 2023-02-25

**Authors:** Kincső Decsi, Barbara Kutasy, Géza Hegedűs, Zoltán Péter Alföldi, Nikoletta Kálmán, Ágnes Nagy, Eszter Virág

**Affiliations:** a Department of Plant Physiology and Plant Ecology, Campus Keszthely, Hungarian University of Agriculture and Life Sciences Georgikon, Keszthely, Hungary; b EduCoMat Ltd., Keszthely, Hungary; c Department of Information Technology and Its Applications, Faculty of Information Technology, University of Pannonia, Zalaegerszeg, Hungary; d Institute of Metagenomics, University of Debrecen, Debrecen, Hungary; e Department of Environmental Biology, Campus Keszthely, Hungarian University of Agriculture and Life Sciences Georgikon, Keszthely, Hungary; f Department of Biochemistry and Medical Chemistry, University of Pecs, Medical School, Pecs, Hungary; g Research Institute for Medicinal Plants and Herbs Ltd., Budakalász, Hungary; h Department of Molecular Biotechnology and Microbiology, Institute of Biotechnology, Faculty of Science and Technology, University of Debrecen, Debrecen, Hungary

In the original published version of this article, the authors did not include a sentence in their acknowledgements section that they wished to include. The author wants to add “We express our thanks to Dr. Márta Kiniczky for the development and formulation of Elice16Indures” in the acknowledgment part. The authors apologize for the errors. Both the HTML and PDF versions of the article have been updated to correct the errors.

