# Supplementary material for: Natural immunity stimulation using ELICE16INDURES® plant conditioner in field culture of soybean
Source: Heliyon. 2023 Jan 10;9(1):e12907. doi: 10.1016/j.heliyon.2023.e12907 (PMC9860300; doi:10.1016/j.heliyon.2023.e12907)
Supplement: Multimedia component 4 [file mmc4.docx]

**Table S1.** Biologically active ingredients of ELICE16INDURES^®^

| Latin binomial name | Main active ingredients | Biological activity |
| --- | --- | --- |
| *Melissa officinalis* | citral, caryophyllene, citronellal, geranyl acetate, caryophyllene oxide |  |
| *Carum carvi* | carvone, limonene |  |
| *Cinnamon Ceylanicum* | cinnamaldehyde, o-methoxy **cinnamaldehyde**, coumarin | **fungicide** |
| *Syzygium aromaticum* | eugenol, caryophyllene, **eugenyl acetate** | **antibacterial, antivirulent, antioxidant** |
| *Allium sativum* | diallyl disulfide, diallyl trisulfide, methyl allyl disulfide, vinyldithiine, ajoene, **allicin** | **antimicrobial** |
| *Zingiber officinalis* | zingiberene, farnesene, curcumene |  |
| *Calendula officinalis* | pentacyclic triterpene alcohols and triterpendiol monoesters, faradiol esters esterified with mainly myristic- and palmitic acids |  |
| *Origanum majorana* | bicylic monoterpenes cis- and trans-sabine hydrate |  |
| *Salvia officinalis* | alpha and beta thujone, camphor, cineole |  |
| *Thymus vulgaris* | thymol, p-cymol |  |

**Table S2.** Different expressed genes (DEG)

Mendeley: 10.17632/b2732cn4ts.1/Table1

**Table S3.** Up- and downregulated sequences of DEG

Mendeley: 10.17632/b2732cn4ts.1/Table2

**Table S4.** Heatmap of DEG-derived sequences per treatment (marked in blue are contigs of upregulated sequences related to the defense response process)


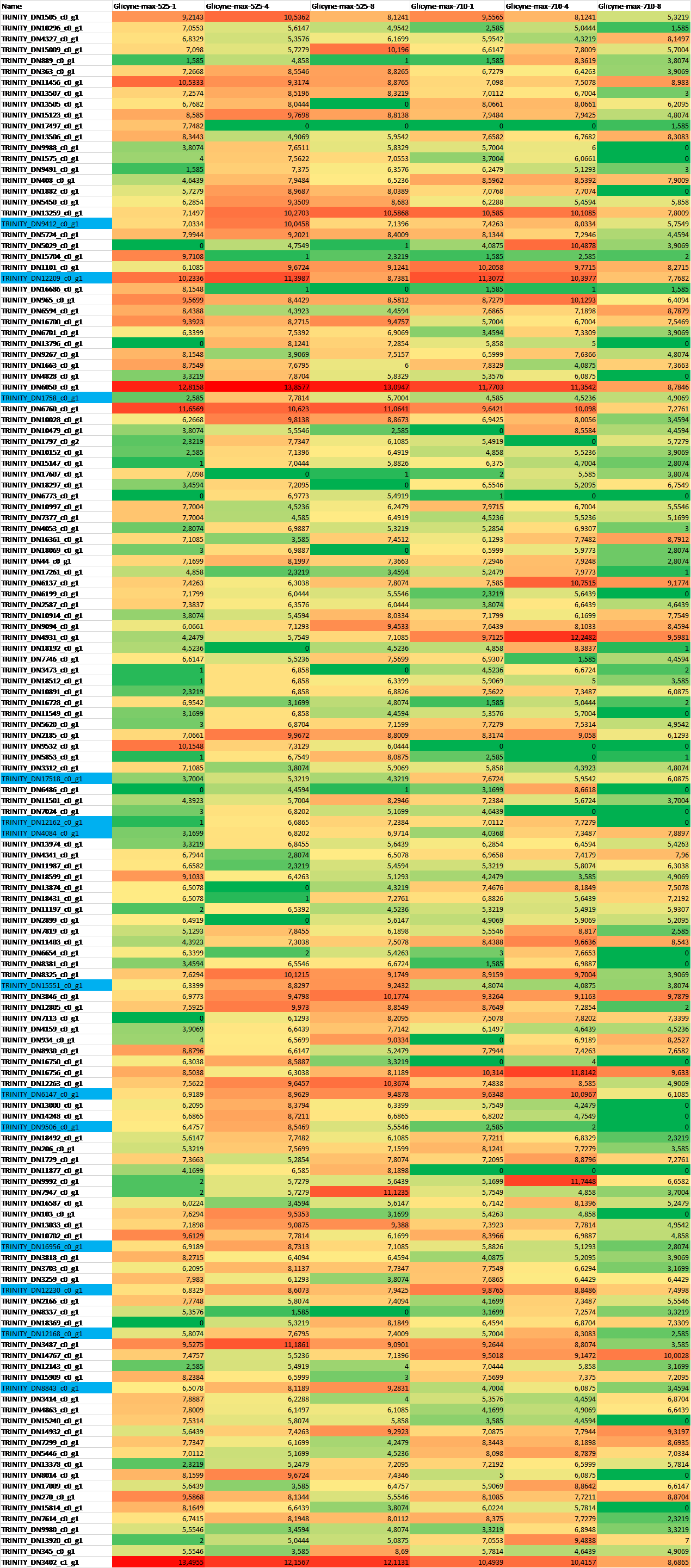


**TableS5**. Gene expression levels in control and treated conditions


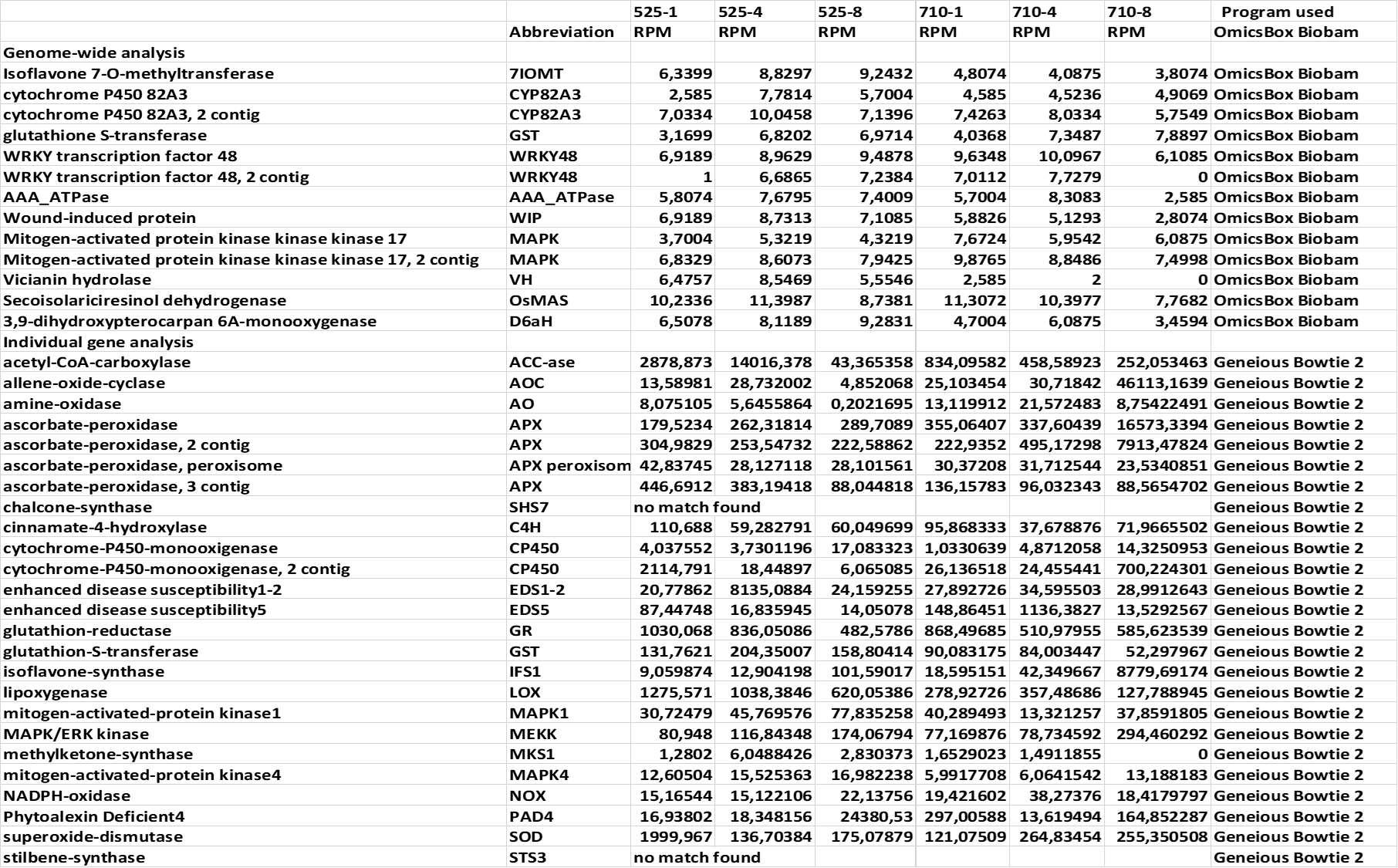


**TableS6.** Primers and conditions of RT-qPCR

| Gene name | Primers | Melting Temperature | Product size |
| --- | --- | --- | --- |
| GAPDH_1 | Left primer: AGTAGATATTTGGCCGTCCTCT | 58,4°C | 107 bp |
| GAPDH_1 | Right primer: ACCATTAGTTTCGATAAACCACCG | 59,4°C | 107 bp |
| Beta-tubulin (TUBB3) | Left primer: GAGCTTGGTGTGGAGAATGGA | 60°C | 101 bp |
| Beta-tubulin (TUBB3) | Right primer: AGCAAGCCAATATAAGAACAAACAGA | 59,6°C | 101 bp |
| AOC | Left primer:  TTGTGGGTCAAGATGTCAGGT | 59,2°C | 101 bp |
| AOC | Right primer:  ATTCCCATCTCCATCACTGCA | 59,2°C | 101 bp |
| GST | Left primer:  TCATCGAGTAGGTTTCATGTTGGA | 59,8°C | 120 bp |
| GST | Right primer: GCATGAAGTTTGGCACATCCA | 59,7°C | 120 bp |
| IFS | Left primer: AGAAGCCCCTTGACTTGACC | 59,6°C | 101 bp |
| IFS | Right primer: GCGAGCGATGTCTCTGATCT | 59,7°C | 101 bp |
| MAPK4 | Left primer: GCGCACAGAAGATATGAAAACGTAT | 60,3°C | 126 bp |
| MAPK4 | Right primer:  TCTTTCCTCAGTACATCTCAACAA | 57,6°C | 126 bp |
| MEKK | Left primer: GTGTTGTTCCTGCTGAGAGC | 59,1°C | 140 bp |
| MEKK | Right primer: TCCACCACCGAAACCCTAAC | 59,6°C | 140 bp |
